# Efficacy of BEAM versus BeEAM conditioning regimens in autologous hematopoietic stem cell transplantation for diffuse large B-cell lymphoma: a retrospective single-center study

**DOI:** 10.3389/fonc.2026.1730912

**Published:** 2026-08-03

**Authors:** Saibiya Akebaer, Gang Chen, Jianli Xu, Kaile Zhang, Ruixue Yang, Chunxia Han, Jia Hou, Ming Jiang, Hailong Yuan

**Affiliations:** Hematology Center, the First Affiliated Hospital of Xinjiang Medical University, Xinjiang Institute of Hematology, Urumqi, China

**Keywords:** diffuse, hematopoietic stem cell transplantation, large B-cell, lymphoma, prognosis, transplantation conditioning

## Abstract

The BEAM regimen (carmustine, etoposide, cytarabine, melphalan) is the standard conditioning regimen for autologous hematopoietic stem cell transplantation (auto-HSCT) in diffuse large B-cell lymphoma (DLBCL). Due to global carmustine shortages, bendamustine-based BeEAM has been proposed as an alternative. This study compared clinical outcomes between BEAM and BeEAM regimens in DLBCL patients undergoing auto-HSCT. A retrospective analysis of 60 consecutive DLBCL patients undergoing auto-HSCT (2019–2024) was conducted. Patients received either BEAM (n=45) or BeEAM (n=15) conditioning. Endpoints included progression-free survival (PFS), overall survival (OS), and treatment response. Survival outcomes were analyzed using the Kaplan-Meier method and the log-rank test. The BEAM group demonstrated significantly superior 3-year PFS (88.7% vs. 75.8%, p=0.024) and OS (95.3% vs. 68.9%, p=0.017) compared to BeEAM. In consolidation patients (n=50), BEAM showed numerically better PFS (89.5% vs. 81.8%, p=0.25) and OS (94.5% vs. 80.8%, p=0.069). Among patients with a pre-transplant complete response (n=52), BEAM showed significantly improved 3-year OS (94.2% vs. 68.9%, p=0.04) and numerically better PFS (86.1% vs. 75.8%, p=0.059). All patients engrafted successfully with comparable neutrophil and platelet recovery. The complete response rate improved from 86.7% (52/60) pre-transplant to 100% (60/60) post-transplant. In summary, BEAM may be associated with better outcomes than BeEAM in this cohort. However, due to the limited sample size, these findings should be interpreted as exploratory, and larger prospective studies are needed to validate these observations.

## 1 Introduction

Diffuse large B-cell lymphoma (DLBCL) represents the most common subtype of non-Hodgkin lymphoma (NHL), with high-dose chemotherapy autologous hematopoietic stem cell transplantation (auto-HSCT) remaining the standard treatment option (1, 2). Auto-HSCT demonstrates a favorable safety profile (with transplant-related mortality rates ranging from 1% to 5%) and has shown significant improvements in overall survival (OS) and progression-free survival (PFS) compared to conventional salvage chemotherapy regimens (3). The myeloablative conditioning regimens before auto-HSCT aim to overcome lymphoma cell resistance and remove minimal residual disease, forming the cornerstone of this approach (4). BEAM (carmustine, etoposide, cytarabine, and melphalan), widely adopted since 1990 with well-established safety and efficacy profiles, remains the most classical and commonly used conditioning regimen for auto-HSCT in relapsed or refractory lymphoma patients (5–7).

In recent years, global production shortages and limited supply of carmustine have prompted the exploration of bendamustine as a promising alternative, leading to the development of the BeEAM regimen (bendamustine-replaced BEAM) for NHL (8, 9). The BeEAM regimen was first proposed by Visani and colleagues in 2011, representing a landmark innovation that substituted carmustine with bendamustine to mitigate carmustine-specific organ toxicities, particularly pulmonary injury, while maintaining cytotoxic efficacy (10). A subsequent update from the same group confirmed a 3-year PFS of 72% in resistant lymphoma patients (11). Bendamustine is a bifunctional alkylating agent that combines the alkylating activity of a mustard group with the antimetabolite properties of a purine analog (12). It additionally induces DNA damage stress responses, promotes apoptosis, suppresses mitotic checkpoints in tumor cells, and activates p53-dependent stress pathways (13). Multiple studies have confirmed the safety and efficacy of bendamustine as a substitute for carmustine (14–17). Similarly, the limited availability of carmustine in China has led our center to adopt bendamustine replacement during auto-HSCT procedures. As BeEAM represents a relatively novel conditioning regimen, its therapeutic efficacy requires further comprehensive evaluation.

However, existing comparative studies on BEAM versus BeEAM have yielded conflicting results (1, 18). Some studies have reported comparable survival and toxicity profiles between the two regimens (1, 13), while others have raised concerns regarding higher incidences of mucositis, febrile neutropenia, or viral reactivation associated with BeEAM (17). These discrepancies, along with variations in bendamustine dosing schedules and patient populations, highlight the need for further real-world evidence to guide regimen selection (4).

This study aimed to compare clinical outcomes between DLBCL patients receiving either BeEAM or conventional BEAM conditioning followed by auto-HSCT. The primary outcomes were PFS and OS, while the secondary outcome was post-transplant treatment response. This research provides urgently needed clinical evidence to guide conditioning regimen selection in resource-limited settings and contributes to optimizing transplant strategies for DLBCL patients.

## 2 Methods

### 2.1 Study participants

This was a single-center retrospective cohort study. Consecutive sampling was used. A total of 60 consecutive DLBCL patients who underwent auto-HSCT at the Bone Marrow Transplantation Center of the First Affiliated Hospital of Xinjiang Medical University between March 2019 and July 2024 were enrolled. Patients receiving the BEAM regimen were transplanted between 2019 and 2024, whereas those receiving the BeEAM regimen were transplanted between 2021 and 2024. No *a priori* sample size calculation was performed, as this was an exploratory study. Inclusion criteria: 1) Patients were diagnosed according to the World Health Organization Classification of Haematolymphoid Tumors (5th edition) (19); 2) Patients with DLBCL who underwent 4–19 cycles of chemotherapy; 3) Patients with relapsed/refractory DLBCL. Disease staging was performed using the Ann Arbor classification (20), risk stratification using the International Prognostic Index (21), and cell-of-origin classification according to the NCCN Guidelines (2023 version) (22). Exclusion criteria included: 1) Patients with non-DLBCL subtypes or primary central nervous system DLBCL; 2) Patients declining auto-HSCT; 3) Patients with severe comorbidities. This study received approval from the Ethics Committee of Xinjiang Medical University (Approval No.: K202501-37), and all participants provided written informed consent.

### 2.2 Pre-transplant induction therapy

The induction therapy before transplantation included R-CHOP (rituximab, doxorubicin, vincristine, cyclophosphamide, prednisone), R-CDOP (rituximab, cyclophosphamide, vincristine, liposomal doxorubicin, prednisone), R-EPOCH (rituximab, etoposide, vincristine, doxorubicin, cyclophosphamide, prednisone), R-CHP (rituximab, cyclophosphamide, doxorubicin, prednisone), R-hyper-CVAD regimens A (cyclophosphamide, vincristine, doxorubicin, dexamethasone) and B (rituximab, methotrexate, cytarabine), and R-MTX-Ara (rituximab, high-dose methotrexate, cytarabine).

### 2.3 Mobilization, collection, and cryopreservation of autologous hematopoietic stem cells

All 60 patients underwent peripheral blood stem cell mobilization. The mobilization protocols included: CHOP-E (cyclophosphamide, doxorubicin, vincristine, etoposide), CDOP-E (cyclophosphamide, liposomal doxorubicin, vincristine, etoposide), EPOCH (etoposide, vincristine, doxorubicin, cyclophosphamide, prednisone), granulocyte colony-stimulating factor (G-CSF) plus plerixafor, and DECP (doxorubicin, etoposide, cisplatin, prednisone). Following collection, autologous hematopoietic stem cells were suspended in a cryoprotective medium containing 10% dimethyl sulfoxide, aliquoted, and cryopreserved at -80 °C. The target CD34^+^ cell count for a single auto-HSCT procedure was optimally greater than 2×10^6^ cells/kg.

### 2.4 Conditioning regimens

Before auto-HSCT, the BEAM and BeEAM conditioning regimens were performed. The standard BEAM conditioning regimen included carmustine (300 mg/m^2^, intravenous infusion, days -8 and -7), etoposide (100 mg/m^2^, intravenous infusion, every 12 hours, days -6 to -3), cytarabine (100 mg/m^2^, intravenous infusion, every 12 hours, days -6 to -3), and melphalan (140 mg/m^2^, day -2). The BeEAM regimen consisted of bendamustine (100 mg/m^2^, intravenous infusion, days -8 and -7), etoposide (100 mg/m^2^, intravenous infusion, every 12 hours, days -6 to -3), cytarabine (100 mg/m^2^, intravenous infusion, every 12 hours, days -6 to -3), and melphalan (140 mg/m^2^, day -2). During the conditioning period, all patients received prophylactic treatments for oral mucositis, diarrhea, and hepatic sinusoidal obstruction syndrome. Patients with comorbidities received corresponding supportive therapies.

### 2.5 Follow-up and efficacy evaluation

Patients were followed through outpatient visits, hospital readmissions, social media platforms, and telephone interviews until December 31, 2024, with no loss to follow-up. Patients underwent follow-up positron emission tomography-computed tomography (PET-CT) scans at 2–3 months post-auto-HSCT, followed by contrast-enhanced whole-body CT and superficial lymph node ultrasound every three months. From years 3-5, examinations were conducted every six months and annually after 5 years to monitor disease progression.

Treatment response was assessed according to the Lugano classification criteria (23), incorporating both CT-based radiographic evaluation and PET-CT-based metabolic assessment using the Deauville 5-point scale. Treatment response was categorized per Lugano criteria (23) as complete response (CR), partial response (PR), very good partial response (VGPR), stable disease, or progressive disease. PFS was defined as the time from transplantation to disease progression, relapse, or death. OS was defined as the time from transplantation to death or last follow-up. PFS and OS served as the primary endpoint, while the post-auto-HSCT treatment response was the secondary endpoint.

Treatment-related toxicities were assessed and graded according to the Common Terminology Criteria for Adverse Events (CTCAE) version 5.0 (24). Four toxicities (oral mucositis, febrile neutropenia, nephrotoxicity, and hepatotoxicity) were prospectively monitored. The highest grade of each toxicity during the post-transplant period (from conditioning initiation to day +100) was recorded for each patient. Assessments were performed daily during hospitalization, then at least weekly until day +100. Febrile neutropenia was defined as an absolute neutrophil count <0.5×10^9^/L with a single temperature ≥38.3 °C or ≥38.0 °C for more than one hour. Nephrotoxicity and hepatotoxicity were graded based on serum creatinine, transaminases, and bilirubin levels according to CTCAE criteria. Grading was performed by attending physicians, and all grade ≥3 events were verified by a second reviewer.

### 2.6 Statistical analysis

Statistical analyses were performed using SPSS version 26.0. Age was not normally distributed (assessed by the Shapiro-Wilk test) and is therefore presented as median (range) and compared using the Mann-Whitney U test. Categorical variables are presented as counts (percentages) and were compared using the chi-square test or Fisher’s exact test, as appropriate. Survival analysis was conducted using the Kaplan–Meier method, with between-group comparisons performed by the log-rank test. All survival estimates (3-year PFS and OS rates) were reported with their 95% confidence intervals (CIs), which were calculated using the log-log transformation method (R package ‘survival’, conf.type = “log-log”) to avoid invalid boundaries (e.g., negative values or >100%) that may occur with the default linear approximation in small samples. A two-sided P-value < 0.05 was considered statistically significant.

## 3 Results

### 3.1 Patient characteristics and treatment profiles

**Figure 1:**
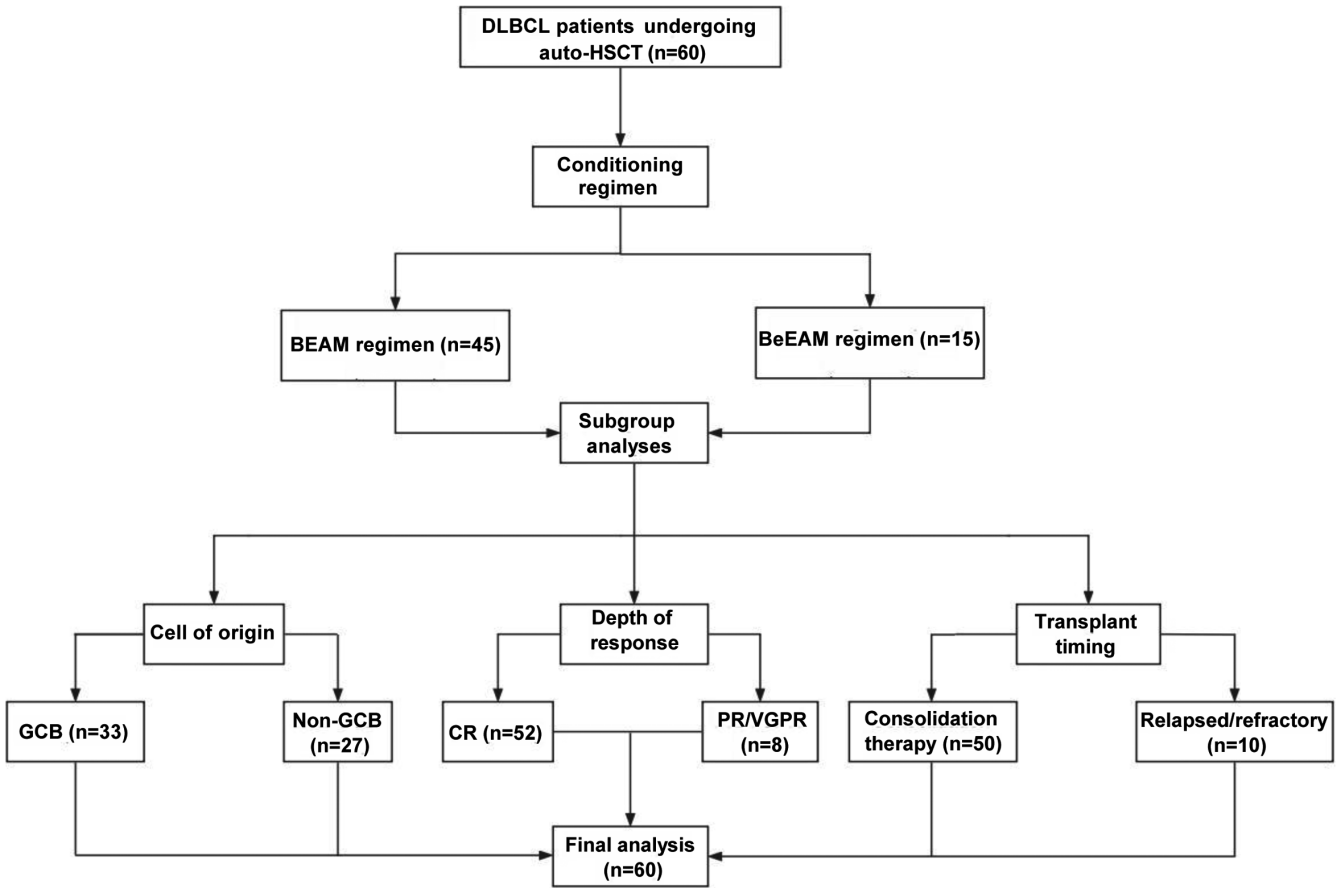
The study flow diagram of patient selection. DLBCL, diffuse large B-cell lymphoma; auto-HSCT, autologous stem cell transplantation; CR, complete remission; PR, partial remission; VGPR, very good partial remission; GCB, germinal center B-cell-like.

The study flow diagram is presented in **Figure 1**. Based on conditioning regimens, patients were divided into BEAM (n=45) and BeEAM (n=15) groups. Baseline characteristics, including age, sex, Ann Arbor stage, IPI score, cell-of-origin subtype, and transplant timing, were well balanced between the two groups (**Table 1**). In the BEAM group, induction regimens included R-CHOP (n=14), R-CDOP (n=12), R-EPOCH (n=16), R-CHP (n=1), R-hyper-CVAD A/B (n=1), and R-MTX-Ara (n=1); in the BeEAM group: R-CHOP (n=7), R-CDOP (n=4), and R-EPOCH (n=4). The median number of induction cycles was 6.5 (range: 4–19) overall. Mobilization protocols for the BEAM group included CHOP-E (n=16), CDOP-E (n=9), EPOCH (n=7), G-CSF plus plerixafor (n=5), and DECP (n=8); for the BeEAM group: CHOP-E (n=5), CDOP-E (n=5), EPOCH (n=1), and G-CSF plus plerixafor (n=4).

**Table 1:** Baseline characteristics of DLBCL patients in the BEAM and BeEAM groups.

Variable	BEAM (n=45)	BeEAM (n=15)	P-value
Median age, years (range)	49 (25–68)	60 (25-68)	0.844
Gender, n (%)			
Male	23 (51.2%)	10 (66.6%)	0.39
Female	22 (48.8%)	5 (33.3%)	0.048
Ann-Arbor stage, n (%)			
II	2 (4.4%)	0	0.57
III	5 (11.2%)	2 (13.3%)	0.76
IV	38 (84.4%)	13 (86.6%)	0.79
B symptoms, n (%)	17 (37.7%)	4 (26.6%)	0.41
Cell of origin, n (%)			
GCB	26 (57.7%)	7 (46.7%)	0.28
Non-GCB	19 (42.3%)	8 (53.3%)	0.37
Risk stratification, n (%)			
Non-high-risk (IPI 0–2)	6 (13.3%)	0	0.18
High-risk group (IPI 3–5)	39 (86.7%)	15 (100%)	0.34
Pre-transplant depth of response, n (%)			
PR	7 (15.6%)	0	0.17
VGPR	1 (2.2%)	0	1.00
CR	37 (82.2%)	15 (100%)	0.34
Transplant timing, n (%)			
Consolidation therapy	39 (86.6%)	11 (73.3%)	0.19
Relapsed/refractory	6 (13.4%)	4 (26.6%)	0.23

Continuous variable (age) is presented as median (range) and was compared using the Mann-Whitney U test (non-normally distributed by Shapiro-Wilk test). Categorical variables are presented as n (%) and were compared using the chi-square or Fisher’s exact test. P < 0.05 was considered statistically significant.

DLBCL, diffuse large B-cell lymphoma; BEAM, carmustine, etoposide, cytarabine, melphalan; BeEAM, bendamustine, etoposide, cytarabine, melphalan; IPI, International Prognostic Index; GCB, germinal center B-cell-like; CR, complete response; PR, partial response; VGPR, very good partial response.

### 3.2 Engraftment and hematopoietic recovery

All 60 patients achieved successful engraftment of autologous hematopoietic stem cells. The median mononuclear cell count infused was 10.72 × 10^8^/kg (range: 4.59–18.38 × 10^8^/kg), and the median CD34^+^ cell count was 7.68 × 10^6^/kg (range: 1.32–23.70 × 10^6^/kg). Hematopoietic recovery was confirmed in all patients post-transplantation. The median time to neutrophil engraftment (defined as the first of three consecutive days with neutrophils > 0.5 × 10^9^/L) was 11.7 days (range: 9–20 days). The median time to platelet engraftment (defined as the first of seven consecutive days with platelets > 20 × 10^9^/L without transfusion support) was 18.3 days (range: 7–51 days).

### 3.3 Treatment-related toxicities

Treatment-related toxicities were compared between the two groups, as shown in **Table 2**. The incidences of oral mucositis, febrile neutropenia, and nephrotoxicity were comparable between the BEAM and BeEAM groups, with no statistically significant differences (P > 0.05 for all comparisons). No severe hepatotoxicity (grade ≥3) was observed in either group.

**Table 2:** Comparison of treatment-related toxicities between BEAM and BeEAM groups.

Toxicity	BEAM (n=45)	BeEAM (n=15)	P-value
Oral mucositis, n (%)	9 (20.0)	4 (26.7)	0.72
Febrile neutropenia, n (%)	22 (48.9)	8 (53.3)	0.76
Nephrotoxicity (any grade), n (%)	6 (13.3)	3 (20.0)	0.65

P-values were calculated using Fisher’s exact test.

BEAM, carmustine, etoposide, cytarabine, melphalan; BeEAM, bendamustine, etoposide, cytarabine, melphalan.

### 3.4 Depth of response post-transplantation

Treatment response was evaluated in all 60 patients before and after auto-HSCT using the Lugano classification criteria (23). Pre-auto-HSCT assessments revealed CR in 52 patients (86.7%), VGPR in 1 patient (1.7%), and PR in 7 patients (11.7%). Post-auto-HSCT, the patient with VGPR and all 7 patients with PR achieved CR, resulting in a CR rate of 100% (60/60), representing a 13.3% increase from pre-auto-HSCT levels.

### 3.5 Follow-up and survival outcomes

As of the end of December 2024, the median follow-up duration for the 60 DLBCL patients was 34 months (range: 4–69 months). The median follow-up was 36.0 months for the BEAM group and 15.0 months for the BeEAM group. Post-auto-HSCT, 8 patients experienced relapse, including 6 with peripheral recurrence and 2 with central nervous system involvement. Five patients died, including 3 due to relapse-related causes and 2 from non-relapse events (1 COVID-19-associated death and 1 death from multidrug-resistant *Pseudomonas aeruginosa* infection). Specifically, the BEAM group reported five cases of relapse or death, while the BeEAM group had four such cases.

**Figure 2:**
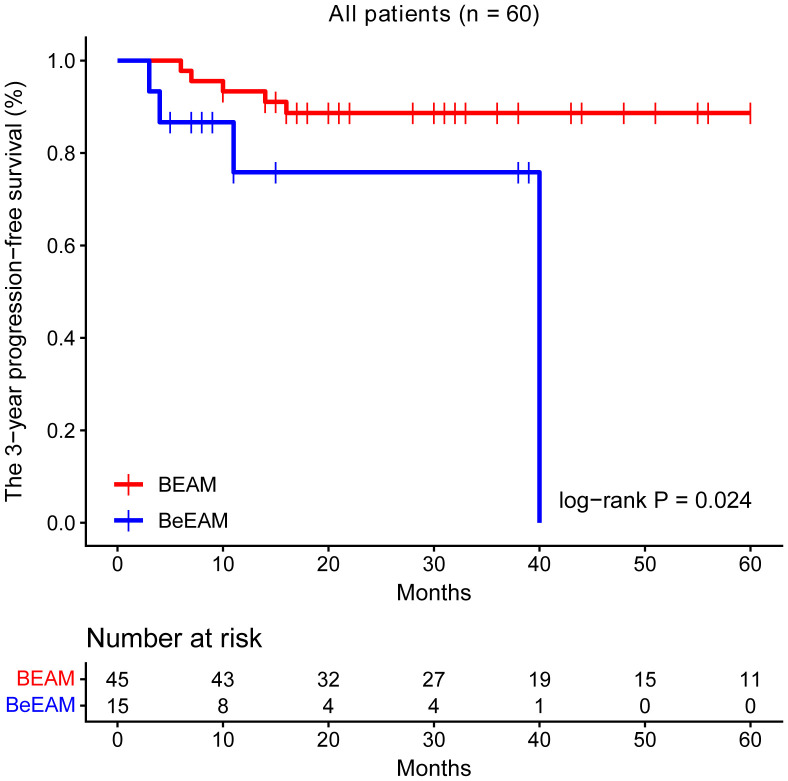
The 3-year PFS rate in all patients. Survival analysis was conducted using the Kaplan-Meier method, with comparisons between the BEAM and BeEAM groups performed by the Log-rank test.

**Figure 3:**
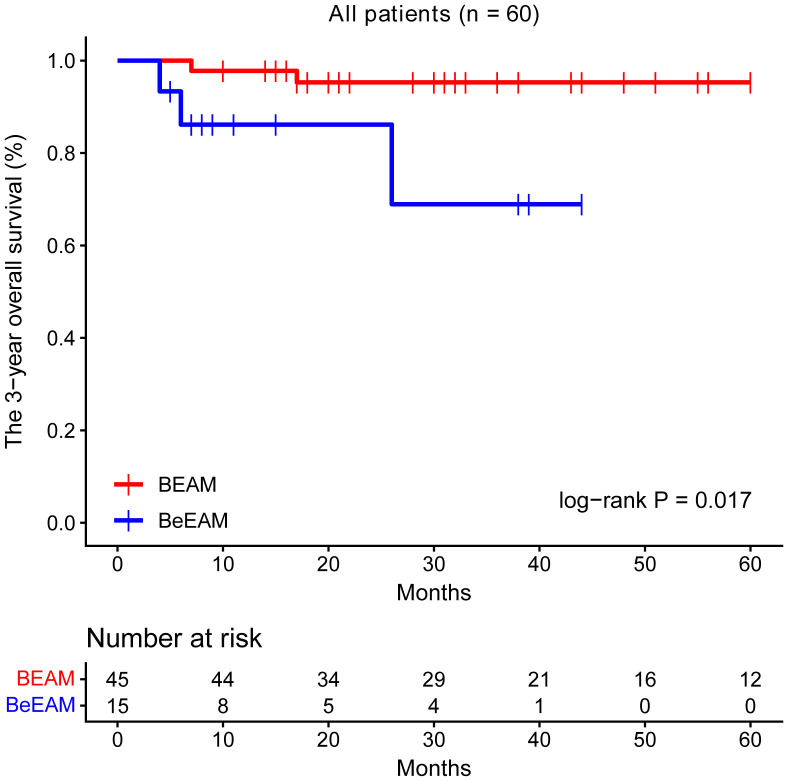
The 3-year OS rate in all patients. Survival analysis was performed using the Kaplan-Meier method, and the difference between the BEAM and BeEAM groups was assessed using the log-rank test.

The 3-year PFS rate was 88.7% (95% CI: 74.90%–95.10%) for the BEAM group, significantly higher than the 75.8% (95% CI: 40.40%–91.90%) for the BeEAM group (p=0.024; **Figure 2**). The 3-year OS rate was 95.3% (95% CI: 82.30%–98.8%) for the BEAM group (2 deaths) and 68.9% (95% CI: 51.27%–86.64%) for the BeEAM group (3 deaths), with a statistically significant difference (p=0.017; **Figure 3**).

### 3.6 Consolidation subgroup analysis

**Figure 4:**
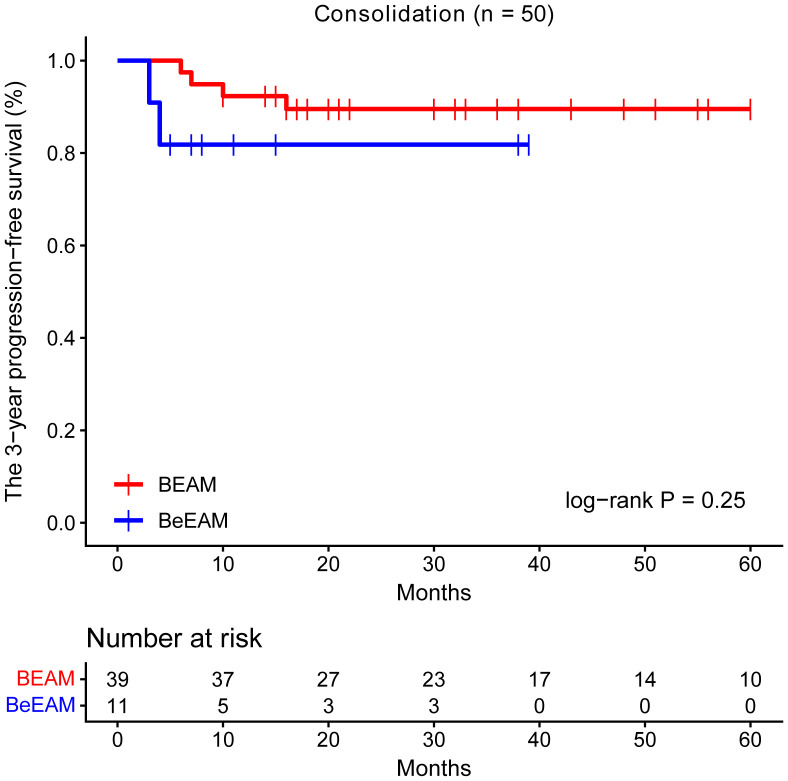
The 3-year PFS rate in patients receiving consolidation auto-HSCT. Survival analysis was conducted using the Kaplan-Meier method, with comparisons between the BEAM and BeEAM groups performed by the Log-rank test.

**Figure 5:**
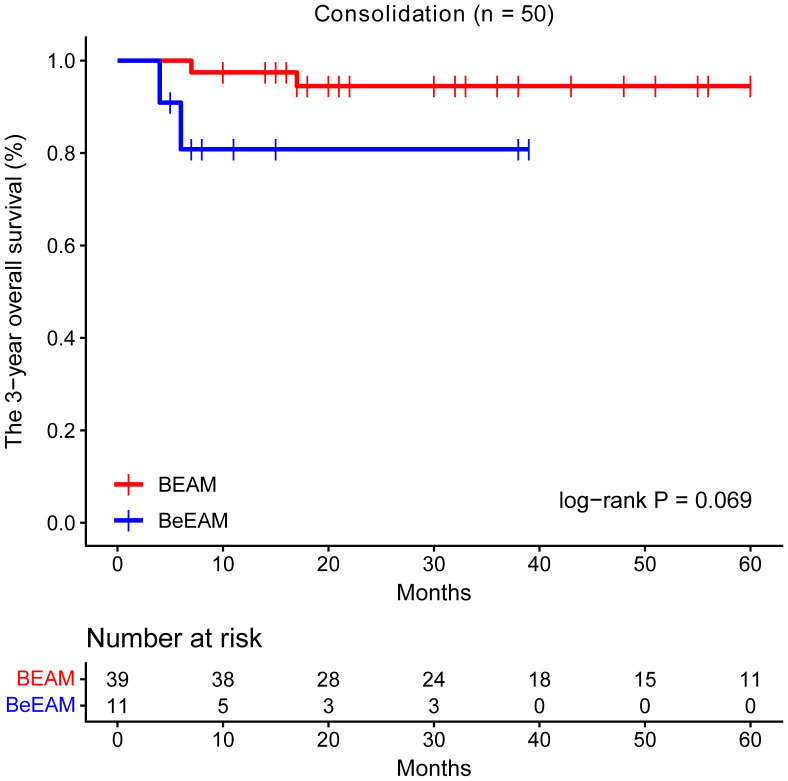
The 3-year OS rate in patients receiving consolidation auto-HSCT. The Kaplan-Meier method was used for survival analysis. The difference between the BEAM and BeEAM groups was compared by the Log-rank test.

Patients were stratified by transplant timing into consolidation auto-HSCT (n=50) and relapsed/refractory disease (n=10). Among consolidation patients, 39 received the BEAM regimen and 11 received the BeEAM regimen. The 3-year PFS rate was 89.5% (95% CI: 74.4%–95.9%) for the BEAM subgroup and 81.8% (95% CI: 44.7%–95.1%) for the BeEAM subgroup, with no statistically significant difference (p=0.25; **Figure 4**). The 3-year OS rate was 94.5% (95% CI: 79.6%–98.6%) for the BEAM subgroup and 80.8% (95% CI: 42.3%–94.9%) for the BeEAM subgroup, also without statistical significance (p=0.069; **Figure 5**).

Among the 10 patients who underwent auto-HSCT for relapsed/refractory disease, 6 received the BEAM regimen and 4 received the BeEAM regimen. Descriptively, the 3-year PFS rate was 83.3% for the BEAM subgroup compared to 66.7% for the BeEAM subgroup, and the 3-year OS rate was 100% versus 50.0%. Due to the small sample size, these results are reported for completeness but should be interpreted with caution.

### 3.7 Effect of conditioning regimens and response depth on PFS and OS

**Figure 6:**
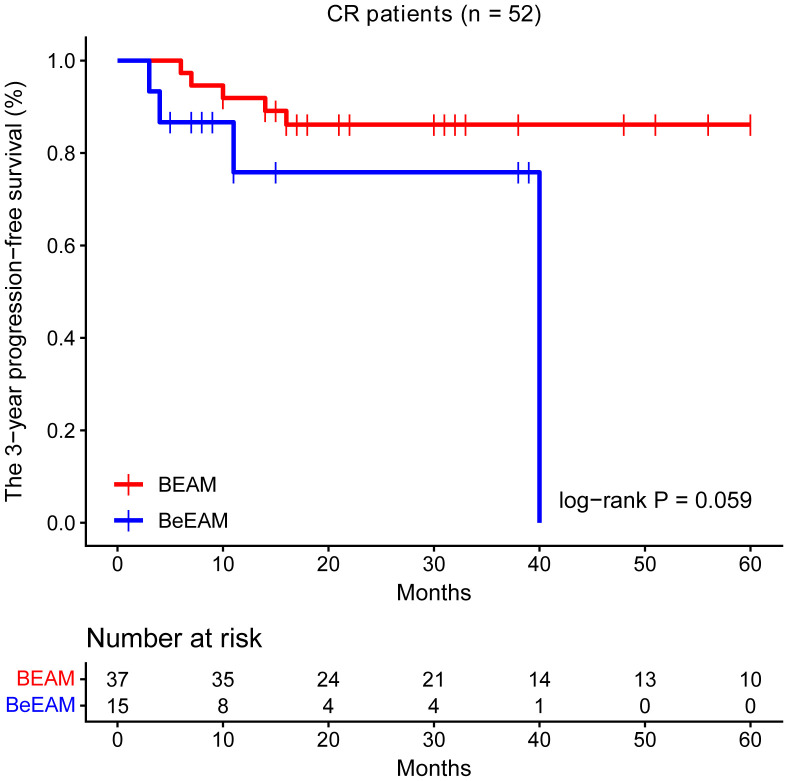
The 3-year PFS rate in patients with CR before auto-HSCT. Survival analysis was conducted using the Kaplan-Meier method, with comparisons between the BEAM and BeEAM groups performed by the Log-rank test.

**Figure 7:**
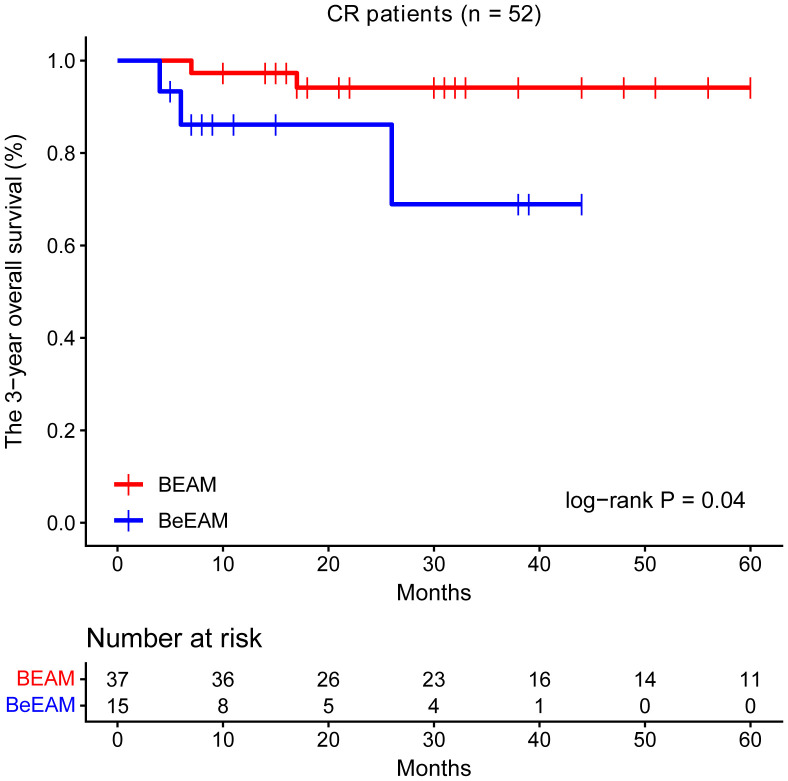
The 3-year OS rate in patients with CR before auto-HSCT. The Kaplan-Meier method was used for survival analysis. The difference between the BEAM and BeEAM groups was compared by the Log-rank test.

Patients were stratified by pre-transplant response depth into CR (n=52) and PR/VGPR (n=8) groups. Among CR patients, 37 received the BEAM regimen and 15 received the BeEAM regimen. The 3-year PFS rate was 86.1% (95% CI: 69.80%–94.0%) for the BEAM subgroup versus 75.8% (95% CI: 40.40%–91.90%) for the BeEAM subgroup. Although numerically superior, this difference did not reach statistical significance (p=0.059; **Figure 6**). However, the 3-year OS rate was significantly higher in the BEAM subgroup (94.2%, 95% CI: 78.50% – 98.50%) compared to the BeEAM subgroup (68.9%, 95% CI: 25.4%–90.4%) (p=0.04; **Figure 7**).

### 3.8 Subgroup analyses by cell-of-origin

Patients were categorized by cell-of-origin into GCB (33 patients) and non-GCB (27 patients) subtypes. Among GCB-subtype patients, 26 received the BEAM regimen and 7 received the BeEAM regimen. Descriptively, the 3-year PFS rate was 88.3% for the BEAM group compared to 80.0% for the BeEAM group, and the 3-year OS rate was 91.8% versus 80.0%. Among non-GCB patients, 19 received the BEAM regimen and 8 received the BeEAM regimen. Descriptively, the 3-year PFS rate was 89.5% for the BEAM group compared to 77.8% for the BeEAM group, and the 3-year OS rate was 100.0% versus 72.9%. Due to the small sample sizes (BeEAM, n=7 for GCB; n=8 for non-GCB), these results are reported for completeness but should be interpreted with caution.

## 4 Discussion

The conditioning regimen plays a key role in auto-HSCT outcomes. While the carmustine-based BEAM regimen remains the standard choice, global supply shortages and pulmonary toxicity concerns have prompted interest in bendamustine-based BeEAM as an alternative (25). Several studies have compared these regimens. Redondo et al. (26) reported 3-year PFS/OS of 58%/75% in 60 NHL patients (38 DLBCL) receiving BeEAM. Krawiec et al. (17) found comparable PFS between BEAM and BeEAM in 82 HL/NHL patients, with differing toxicity profiles (higher gastrointestinal toxicity with BeEAM, higher pneumonitis with BEAM). Hahn et al. (27) also reported non-significant differences in 3-year PFS (71.3% vs. 74.1%) and OS (78.1% vs. 71%) between the two regimens. In contrast, our findings suggest that BEAM may offer superior outcomes compared to BeEAM in DLBCL patients. This discrepancy may reflect differences in patient population (exclusively DLBCL in our study versus mixed subtypes in others), bendamustine dosing, or supportive care strategies.

Auto-HSCT remains a key strategy for high-risk lymphoma, serving as both consolidation and salvage therapy (28). D.F. et al. reported superior 3-year PFS for consolidation versus salvage auto-HSCT (93.3% vs. 58.7%) with BEAM conditioning (29). Garcia-Sancho et al. (30) reported favorable 5-year PFS (63%) and OS (74%) with BEAM in peripheral T-cell lymphoma. For BeEAM, Noesslinger et al. (31) reported 4-year PFS/OS of 55.6%/72.6% in 41 lymphoma patients. Lachance et al. similarly reported the feasibility of implementing a bendamustine-based conditioning regimen in clinical practice (4). A randomized phase II trial (n=108) showed comparable 1-year PFS (72.5% vs. 81.7%) and OS (92.3% vs. 88.6%) between BeEAM and BEAM (18). Plante et al. (13) reported 2-year PFS (65%) and OS (83%) in DLBCL patients receiving BeEAM. Our subgroup analysis in consolidation patients showed numerical differences favoring BEAM, whereas the relapsed/refractory subgroup (n=10) was too small for meaningful inference and requires validation in larger studies. Nonetheless, two points are worth noting: consolidation auto-HSCT appears superior to salvage transplantation regardless of regimen, and while BeEAM may be a reasonable alternative when BEAM is unavailable, our data suggest BEAM may be preferred, particularly in patients with aggressive disease features.

The toxicity profiles of the two conditioning regimens were generally similar in our cohort. These findings are consistent with previous reports by Krawiec et al. (17) and Hahn et al. (27), who also found comparable toxicity profiles between BEAM and BeEAM, albeit with some differences in specific toxicities (e.g., higher gastrointestinal toxicity with BeEAM and higher pneumonitis incidence with BEAM). The absence of significant toxicity differences in our study suggests that the observed survival disadvantage of the BeEAM regimen is unlikely to be driven by differential toxicity-related mortality or supportive care requirements.

The depth of pre-transplant response is a well-established prognostic factor in auto-HSCT. Mei et al. (32) reported superior 1-year PFS (69% vs. 48%) and OS (84% vs. 63%) in CR versus PR patients following BEAM/R-BEAM conditioning, with sustained OS benefit at 3 years (64% vs. 50%). Similarly, Redondo et al. (26) observed worse outcomes in PR versus CR patients receiving BeEAM. In our CR subgroup, BEAM was associated with better 3-year OS (94.2% vs. 68.9%) and numerically better PFS (86.1% vs. 75.8%) compared to BeEAM. These findings further reinforce the importance of achieving CR before transplantation.

The prognostic role of cell-of-origin in auto-HSCT outcomes remains debated. Joseph et al. (33) found no significant correlation between cell-of-origin and survival in 122 relapsed/refractory DLBCL patients receiving BEAM, though GCB patients had longer median PFS (44.8 vs. 28.5 months). Another study comparing BEAM versus BeEAM in 60 relapsed/refractory DLBCL patients demonstrated no significant difference in 5-year OS (60% vs. 72.5%) or PFS (20% vs. 25%) between regimens, with balanced cell-of-origin distribution between groups (34). Kim et al. (35) reported comparable 5-year PFS between GCB and non-GCB subtypes in high-risk DLBCL. In our cohort, BEAM showed numerically better outcomes in both subtypes; however, due to the small sample sizes (BeEAM, n=7 for GCB; n=8 for non-GCB), these observations are purely descriptive and require validation in larger studies.

This study has several limitations. First, its retrospective, single-center design may introduce selection bias, and unmeasured confounding cannot be excluded. Second, the sample size is small, particularly the BeEAM group (n=15), limiting statistical power and generalizability; subgroup analyses by age or sex were not feasible. Third, the median follow-up duration was relatively short, and longer-term outcomes and late toxicity profiles require further investigation. Fourth, detailed toxicity comparisons (e.g., pulmonary toxicity, supportive care requirements) and comorbidity data were not systematically collected. These limitations underscore the need for larger, prospective, multi-center studies.

Taken together, this study suggests that BEAM may be associated with better outcomes than BeEAM in this cohort of DLBCL patients undergoing auto-HSCT, with improved PFS and OS observed. In settings where carmustine is unavailable, the BeEAM conditioning regimen represents a feasible alternative. However, due to the limited sample size and small number of events, these findings should be interpreted as hypothesis-generating rather than definitive. Large-scale, multicenter, prospective studies are needed to confirm these observations.

## Data Availability

The raw data supporting the conclusions of this article will be made available by the authors, without undue reservation.
